# The effects of helminth infections on the human gut microbiome: a systematic review and meta-analysis

**DOI:** 10.3389/frmbi.2023.1174034

**Published:** 2023-05-18

**Authors:** Bridgious Walusimbi, Melissa A. E. Lawson, Jacent Nassuuna, David P. Kateete, Emily L. Webb, Richard K. Grencis, Alison M. Elliott

**Affiliations:** ^1^Immunomodulation and Vaccines Programme, Medical Research Council (MRC)/Uganda Virus Research Institute (UVRI) and London School of Hygiene and Tropical Medicine (LSHTM) Uganda Research Unit, Entebbe, Uganda; ^2^Department of Biomedical Science, College of Health Sciences, Makerere University, Kampala, Uganda; ^3^Lydia Becker Institute of Immunology and Inflammation, Faculty of Biology, Medicine and Health, University of Manchester, Manchester, United Kingdom; ^4^MRC International Statistics and Epidemiology Group, Department of Infectious Disease Epidemiology, London School of Hygiene and Tropical Medicine, London, United Kingdom; ^5^Department of Clinical Research, London School of Hygiene and Tropical Medicine, London, United Kingdom

**Keywords:** microbiome, helminths, alpha-diversity, beta-diversity, Shannon index

## Abstract

The gut microbiome is important in shaping human health. One key factor that has been proposed to affect the gut microbiome is helminth infection. Unravelling the association and/or interaction between helminth infections and the gut microbiome may reveal new insights into the mechanisms through which parasitic worms impact the prognosis of infections and diseases. While considerable work has gone into reviewing data on the effect of helminth infection on gut microbiome in animal studies, less attention has been given to this area of research in human studies. This study set out to address this through an exhaustive systematic review of literature. Articles were identified through EMBASE, MEDLINE, Web of Science and Science Direct following a registered protocol (PROSPERO). After assessing methodological quality (ICROMS) and publication bias, a random effects meta-analysis was performed to investigate the overall effect that intestinal parasites can have on the human gut microbiome using alpha- and beta-diversity metrics and adjusting for age, sex and antihelminthic treatment taken by individuals. A total of 19 out of 3466 articles were included in the final meta-analysis. Our results show that helminth infection increases the host bacterial diversity, as well as microbial richness. This work further contributes to the understanding of how the gut microbiome structure changes depends on whether one is infected with helminths or not. It also lays the foundation for future research aimed at establishing how these interactions could explain the disparity in phenotypes such as infection, disease and vaccine responses reported in different regions worldwide.

**Systematic review registration:**

https://www.crd.york.ac.uk/prospero/, identifier CRD42020192182.

## Introduction

Over the millennia, humans have co-evolved with several trillions of microbes, to the extent that these microbes may now be considered as an important organ of the human body (Baquero and Nombela, 2012). The gut microbiota – the community of microorganisms colonizing the gut (Jonsson and Backhed, 2017) – has been shown to be integral in many facets of human health, spanning immunity, nutrition and energy metabolism (Hakansson and Molin, 2011; Zhang et al., 2015). Several lines of evidence have shown that changes in the gut microbiota composition play a central role in the development and progression of disease (Levy et al., 2017) including diseases such as those that are associated with *Clostridium difficile* infection (CDI; (Leslie et al., 2019), Human Immunodeficiency Virus (HIV; (Bandera et al., 2018), Hepatitis B Virus (HBV; (Alharbi et al., 2019; Liu et al., 2019), as well as obesity (Turnbaugh et al., 2009), Inflammatory Bowel Disease (IBD; (Manichanh et al., 2006), psoriatic arthritis (Scher et al., 2015), atopic eczema (Wang et al., 2008), coeliac disease (Schippa et al., 2010) type 1 diabetes and type 2 diabetes (Turnbaugh et al., 2009; de Mello et al., 2017).

**Figure 1:**
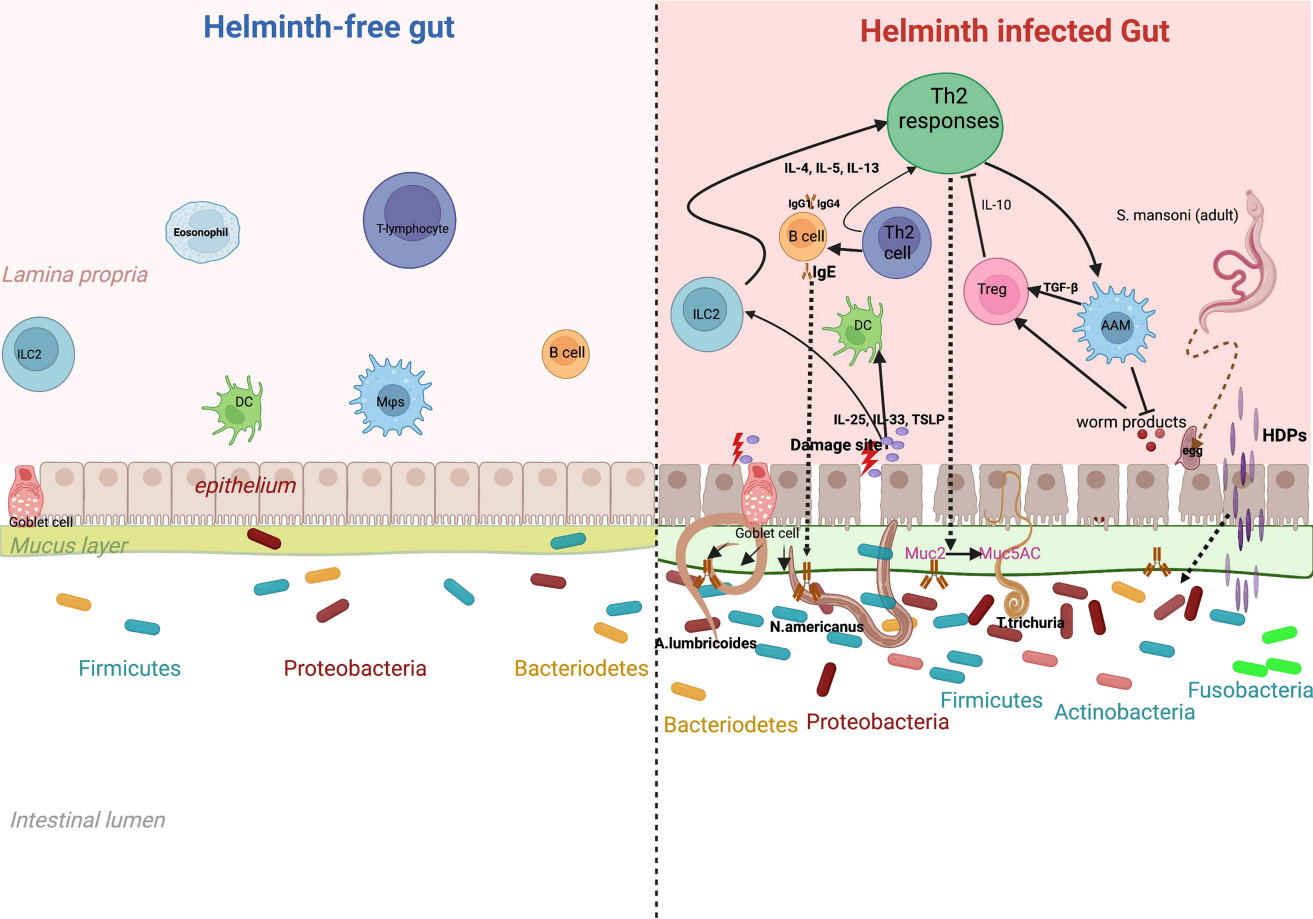
Effect of helminth-induced immune responses on gut microbiota. Chronic helminth infection is associated with a dominant T-helper 2 immune response. This is initiated through alarmins such as IL-25, 1L-33 and thymic stromal lymphopoietin, that are released after tissue damage by the parasite. Alarmins induce production of 1L-4, IL-5 and IL-13 by ILCs that promote induction of Th2 responses. Increased release of IL-4 and IL-5 from CD4+ T cells facilitates production of antibodies IgG, IgE and IgG4 from B-Cells and also activates macrophages (Alternatively Activated macrophages) essential in wound-healing. T-regulatory cells are activated to release anti-inflammatory cytokines such as 1L-10. This marked Th2 response leads to events such as epithelial turnover, goblet cell hyperplasia, increased secretion of mucus (more muc5ac) and Host Defence Peptides (HDPs). These helminth-specific immune responses may explain the microbial differences between a gut infected and one that is free of helminth infection.

Given the effect of gut microbiota on immune responses and disease, it is important to study and understand the various factors that could affect the gut microbiome profile: changes in diet (Walker et al., 2011; David et al., 2014), antibiotic exposure (Jernberg et al., 2007; Jakobsson et al., 2010) and intestinal infections (Manichanh et al., 2006) can have an impact on the abundance and diversity of the microbial species inhabiting the human gut. Furthermore, several studies have consistently reported differences in the gut microbiome between populations living in rural areas compared to those living in urban areas. For example, De Filippo et al. reported that compared to European children, African children had an increased gut bacterial richness that was dominated by Short Chain Fatty Acid (SCFA)-producing *Prevotella* and *Xylanibacter*, which are rarely found in European children (De Filippo et al., 2010). Importantly, another possible contributor to the intestinal microbial variation in humans in this scenario, may be the gut-dwelling helminths. Some helminths (especially soil-transmitted worms) share their habitat (the gut) with dense microbial communities (Houlden et al., 2015; Ramanan et al., 2016), whilst other helminths are more transient through the gut, like *S. mansoni* that lives in the mesenteric vasculature and sheds eggs that move across the intestinal wall to the intestines. While in the gut, helminths can damage the gut epithelium and induce a diverse array of host immune responses, predominantly T-Helper 2 (**Figure 1**) characterized by events such as goblet cell hyperplasia, increased intestinal epithelial turn-over. Also, helminths such as *S. mansoni* secrete Host Defense Peptides (HDPs) that are antimicrobial. On the other hand, a helminth free gut is assumed to maintain a fair level of homeostasis, with an intact epithelium implying that alarmins are not released and as such, the TH2 response here is less pronounced. This background forms the impetus to our review postulating that these differences in the immune responses in presence and absence of helminth infection may have significant potential to impact the gut microbiome composition and diversity in humans (Jenkins et al., 2018).

To decipher the helminth-gut microbiome relationship, a number of studies investigating the impact of parasitic infections on the gut microbiome have been conducted in humans (Easton et al., 2019; Baker-Austin et al., 2020), and more often in animal models (Houlden et al., 2015; Floudas et al., 2019). These show the different associations that helminths may have with the various constituents of the gut microbiota. A recent narrative review by Cantacessi and colleagues highlighted inconsistency in data from studies investigating the effect of the different helminthic parasites, albeit in veterinary species, on several constituents of the microbiome (Cantacessi et al., 2014).

While considerable work has gone into reviewing data on the effect of helminth infection on gut microbiome in animal studies, less research and knowledge is known about what happens in humans. We anticipate that unravelling the association and/or interaction between helminths and the gut microbiome will reveal new insights into the mechanisms that the parasitic worms can impact the prognosis of other infections and/or diseases such as tuberculosis and cardiovascular disease, where helminth infection have been reported to be important (Sanya et al., 2020; Garrido-Amaro et al., 2021).

At the time of generating the protocol for this systematic review and registering it at PROSPERO, there was no such review summarizing the findings on the impact of helminths on the gut microbiome in humans. Since then, a single study has been published (Kupritz et al., 2021) examining how helminths may affect the gut microbiome. However, our current study includes additional aspects like using a random effect model in the meta-analysis and we have included analysis of more human datasets.

## Methods

The protocol used in this review was written in compliance with the meta-analysis of observational studies in epidemiology **(**MOOSE) (Stroup et al., 2000) and Preferred Reporting Items for Systematic Reviews and Meta-Analyses (Shamseer et al., 2015) guidelines, and is registered at the International Prospective Register of Systematic Reviews (PROSPERO) under the ID CRD42020192182.

### Data sources, search terms, and search strategy

The following literature databases were systematically searched for this review: MEDLINE (via PubMed), Web of Science, Science Direct and EMBASE, complemented by Google Scholar literature searches. Search terms used are listed in **Table 1**, briefly they include terms corresponding to four categories: helminths, mucosal associated lymphatic tissues, microbiome and humans.

**Table 1:** Search terms used in literature search.

Helminths	Mucosal associated lymphatic tissues	Microbiome	Humans
parasitic worm*	gastrointestinal	microbes	persons
worms	intestine*	microorganisms	people
intestinal parasites	stomach	bugs	individuals
onchocerca	duodenum	germs	human beings
Tricuris trichiura	bowels	bacteria	
intestinal worms	digestive tract	viruses	
schistosom*	alimentary canal	opportunistic pathogens	
flatworms	gut	commensal	
trematodes		microbio*	
platyhelminthes			
nematodes			
tapeworms			
roundworms			
acanthocephalins			
ascaris			
Ascaris lumbricoides			
hookworm			
Heligmosomoides polygyrus			
Fasciolopsis buski			
opisthorchis			
Clonorchis sinensis			
Ancylostoma duodenale			
Necator americanus			
whipworm			
geohelminths			
threadworms			
dracunculiasis			
mansonella			

Boolean operator ‘OR’ was used to combine the search terms under each of the four categories, and ‘AND’ was used to combine search terms across the four categories to enable the reviewers to collect all published articles and dissertations relevant to the research topic of the planned review. The search performed considered all available literature covering the period up to 10^th^ October 2022.

Duplicates were removed, and the resulting list of unique articles was first screened based on article titles and abstracts. Full-text versions of approved articles which were screened and assessed for inclusion in this review by two independent reviewers following the inclusion and exclusion criteria are described herein. Reference lists from studies that met the inclusion criterion was searched to identify additional studies for consideration in this review.

### Inclusion and exclusion criteria

Following the patient/population, intervention, comparison and outcomes (PICO) strategy, the study inclusion and exclusion criteria for this review are summarized in **Table 2**. This review considered human studies involving helminth infection and/or anthelminthic treatment as the exposure variable and the gut microbiome as the outcome variable. Primary research studies including randomized controlled trials, cross-sectional, cohort and case-control study designs were considered. Articles where helminth infection was diagnosed by standard techniques such as Kato-Katz and Polymerase Chain Reactions (PCRs) were considered for this review. Only articles that were published in English and were conducted in humans were included, microbiome analyses on animal models were excluded.

**Table 2:** Summary of inclusion and exclusion criteria following the PICOS strategy.

PICOS strategy	Inclusion criteria	Exclusion criteria
P –Population	Human participants	Animal models
I-Intervention	With or without treatment involved	
C-comparison	Helminth infected versus uninfected; anthelminthic treatment versus no treatment	No comparison group
O-Outcome	Studies investigating effect of helminths and anthelminthic treatment as exposure and gut microbiome as the outcome	Studies that do not have gut microbiome as the outcome of interest
S-study design	Cohort, cross-sectional, case-control, RCT studies, other Experimental designs	Case series and case reports

### Participants

All identified studies involving human participants, irrespective of age, gender and ethnicity, reported to have either current or previous helminthic infection and/or undertaking anthelminthic treatment, were included in this systematic review and meta-analysis.

### Data extraction

**Figure 2:**
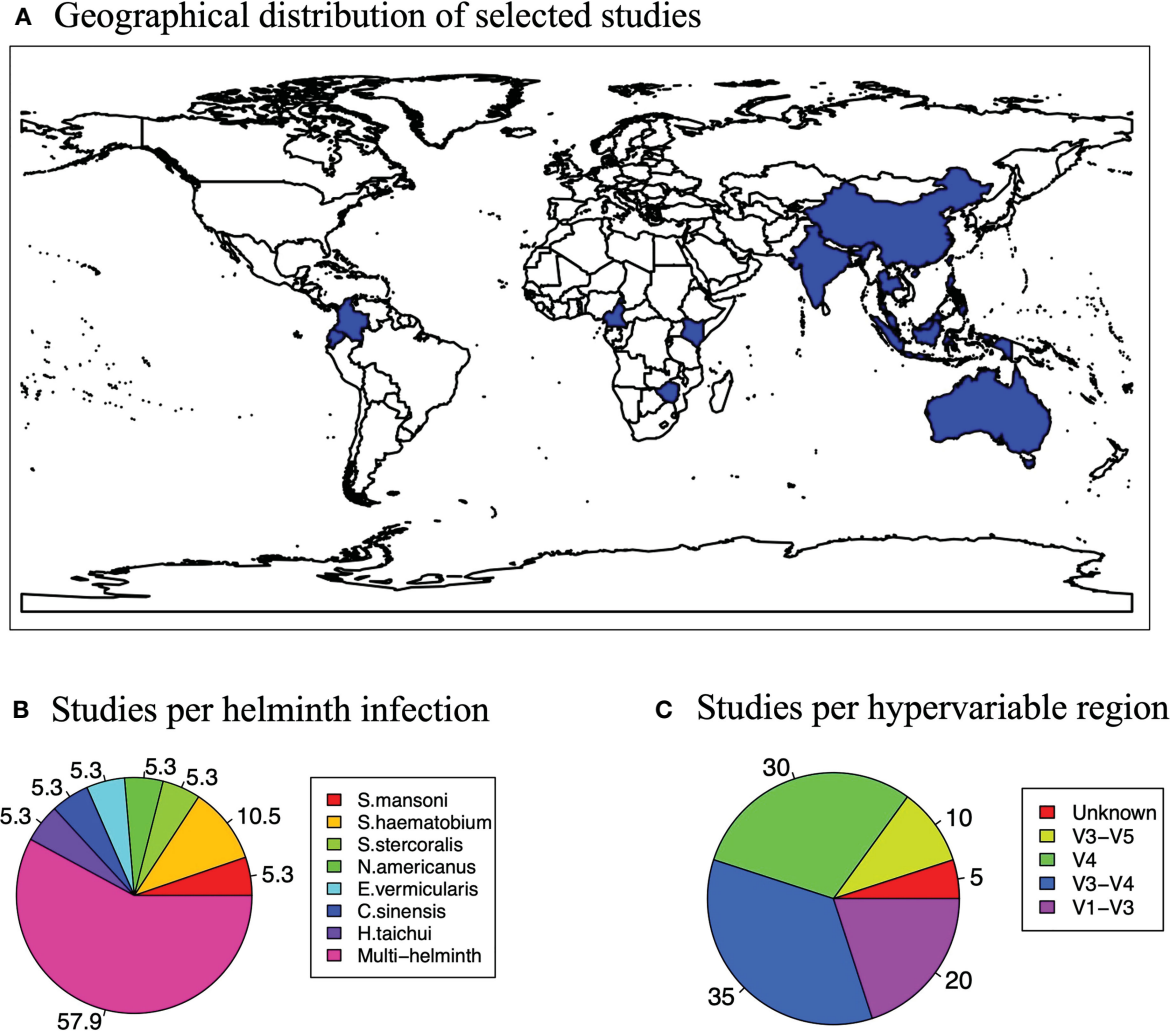
Study characteristics. **(A)** Map showing the global distribution of the studies selected for the systematic review and meta-analysis. **(B)** A pie chart showing how studies varied according to hypervariable regions used for microbiome profiling. **(C)** A pie chart showing how studies varied according to the helminth that participants were infected with in a study.

A data extraction form (Microsoft Excel spreadsheet) adapted from the Cochrane collaboration, designed *a priori*, was used independently by the two reviewers to extract data from the studies that were selected for the review. This extraction tool collected data on study characteristics including information on outcome measures, results, methods used, participants, population and setting, eligibility and general information such as year of publication, country where the study was conducted, the hypervariable region used to profile the microbiome and the sequencing platform used (**Figure 2C**). The reviewers independently extracted the data and then discussed any discrepancies, and a third reviewer was available for consultation in case consensus was not reached.

Assessment of methodological quality (risk of bias). We used the Integrated quality Criteria for Review Of Multiple Study designs (ICROMS) (Zingg et al., 2016) to assess the methodological quality of included articles. This is because studies selected were of various designs including cohort and case-control studies. ICROMS is composed of two parts: a scoring system with criteria of quality assessment that is study design specific, and one that cuts across all study designs. It generates a decision matrix to filter studies depending on whether they meet minimum requirements of inclusion and are also relevant to the planned review question. Following the ICROMS system, studies that met our inclusion criterion fell under Non-Controlled-Before-After (NCBA), Controlled-Before-After (CBA), and Cohort Study (CS) designs. The maximum available score for a NCBA study is 30 points (minimum score required for inclusion in the data synthesis: 18) and for the CBA and CS section, the maximum available score was 28 (minimum score for inclusion in the data synthesis for both CBA and CS: 17, **Supplementary Figure 1**).

### Data analysis

**Figure 3:**
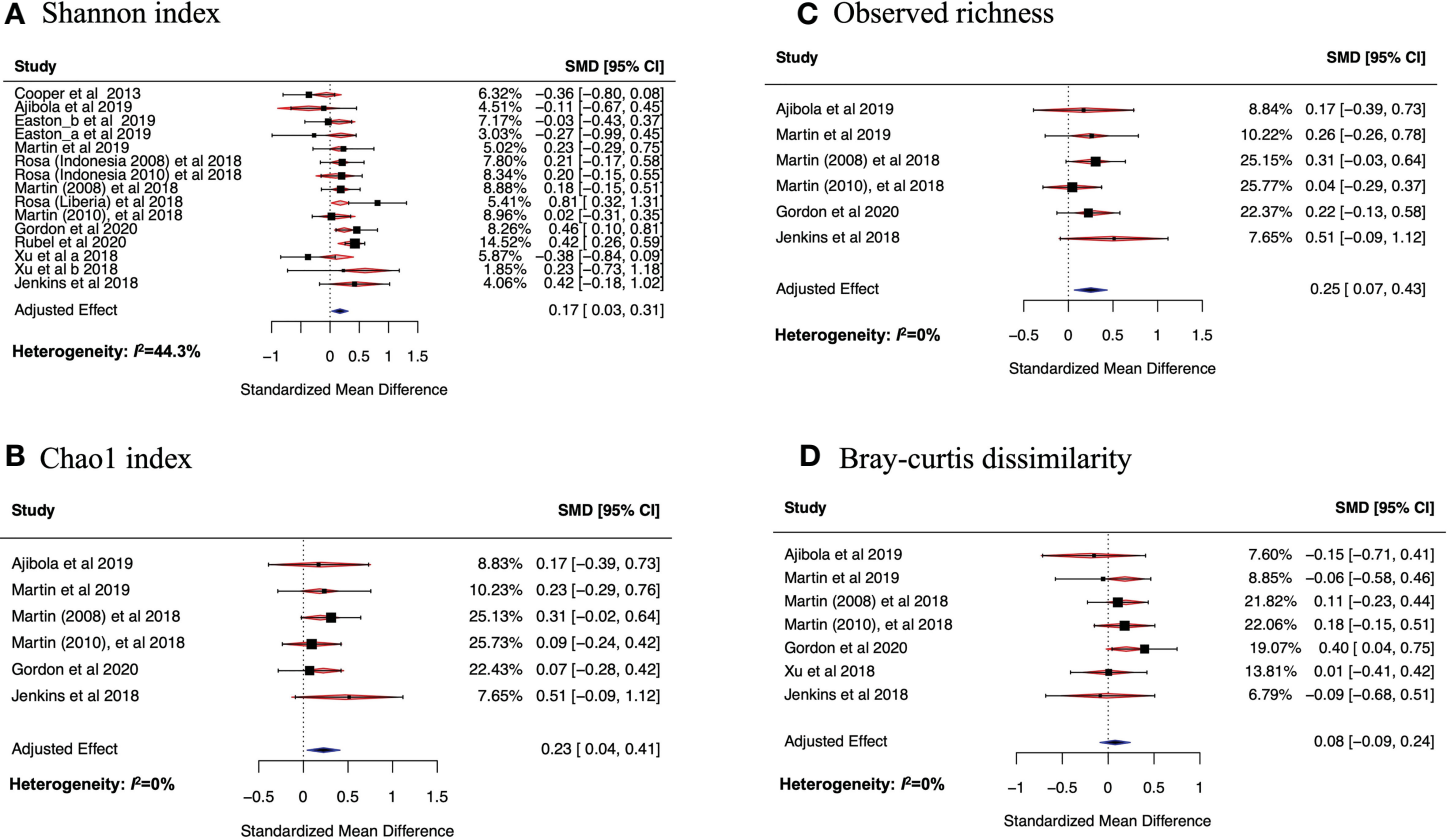
Forest plots of the effect of helminth infections on Shannon **(A)** and Chao1 **(B)** indices, and on observed richness **(C)** and Bray-curtis dissimilarity **(D)**. In **(A)** Easton_a and Easton_b are datasets from the same study but were analyzed differently in the original study because they were from two different batches. The study by Rosa et al. had three datasets. Two of these were from Indonesia but collected at different periods, one (Rosa (Indonesia 2008) et al., 2018) in 2008 and another (Rosa (Indonesia 2010) et al., 2018) in 2010. The third dataset (Rosa (Liberia) et al., 2018 was collected from Liberia. Another study by Martin et al. had two datasets collected in two different time periods: Martin (2008) et al., 2018 was collected in 2008 while Martin (2010) et al., 2018 was collected in 2010. Xu et al. 2018 had two datasets (Xu_a et al.) and Xu_b et al. that belonged to two different batches. These datasets were meta-analyzed using random effects model in **(B-D)**.

Data were mined from **Supplementary Files** of selected papers, public data archives such as Nematode.net and European Nucleotide Archive. For each study included in the meta-analysis, we obtained Operational Taxonomic Units (OTU) tables, taxonomy tables, metadata files that were combined and analyzed in a uniform way using R and Bash to generate both alpha diversity metrics and beta diversity metrics for each of the samples for all the selected studies. Corresponding authors were contacted directly when additional data were required. Alpha-diversity metrics were calculated for each individual participant. For calculation of Bray-Curtis dissimilarity, in each study the participants were grouped into helminth infected and uninfected groups, and the Bray-Curtis dissimilarity was calculated between individuals within these two groups. Averages and their respective standard deviations for Chao1, observed richness, Simpson and Shannon indices were computed for the helminth-infected and helminth-uninfected groups of every study that met the inclusion criterion. Since helminths may have heterogeneous effects on the gut microbiome in humans, we used the random effects model in the meta-analyses. This was performed using the metafor package in R. We used standardized mean differences (SMDs), calculated as the difference in means between the helminth infected and uninfected groups scaled by the standard deviation, as our measure of association with the different diversity metrics for all the datasets. For the BCD, the difference in BCD between groups was used as the measure of association. Funnel plots, along with Egger’s test were performed to test for publication bias in our analysis. To assess the proportion of variation in the estimate of helminth effects due to the heterogeneity between studies, I-squared (*I^2^*) statistic was calculated for each of the metrics and included in each forest plot (**Figure 3**). Data on treatment, age and sex for each study were collected and adjusted for in the analysis.

For papers that reported results from multiple datasets and countries separately, each was included as an individual set of the results in this meta-analysis.

## Results

**Figure 4:**
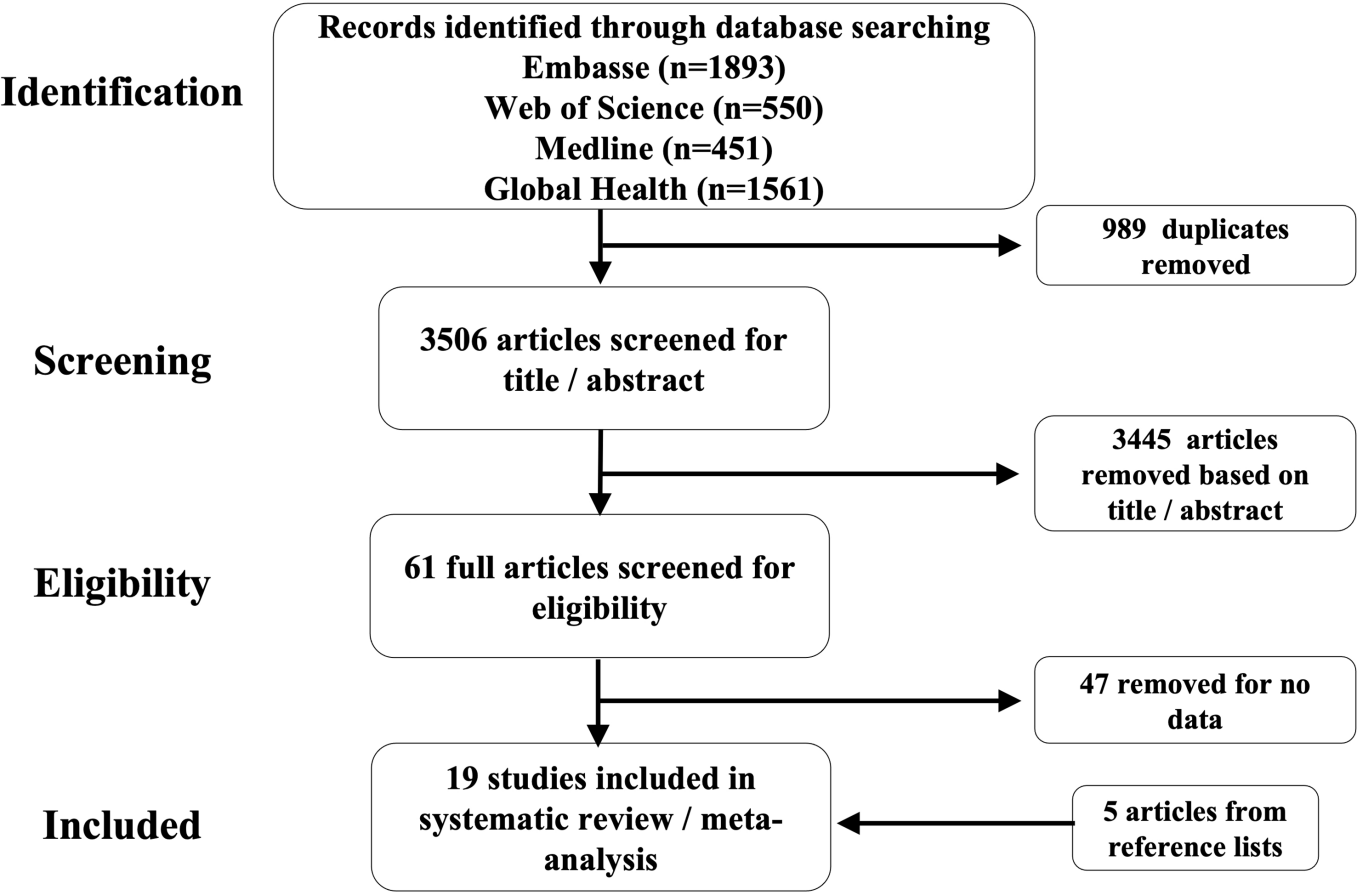
Flow diagram showing the selection process of the studies for the systematic review and metanalysis.

Characteristics of selected articlesOur literature searches yielded 3466 unique articles (excluding duplicate publications). Fourteen articles passed the eligibility criteria and five more articles, relevant to our topic of interest, were added after examining the reference lists of the eligible articles. A total of 19 articles were included in this systematic review as illustrated in **Figure 4**. We were able to obtain 15 datasets from the selected articles for the meta-analysis. The 19 articles (see full list in **Supplementary Figure 2**) that met the inclusion criterion for the systematic review were from 17 countries, and included a total of 1877 participants (847 males and 1030 females) with the smallest sample size as 30 and the largest sample size as 575. The mean age of the participants was 29 years (range 10.3 years to 72 years). The majority of the studies (55.6%) were from China and East Asia while 27.8% were from Sub-Saharan Africa (SSA), 11.1% were from the Americas and 5.5% were from Europe (**Figure 2A**).

### The 16S hypervariable regions targeted for microbial profiling

All the studies included in this review and meta-analysis were based on 16S rRNA sequencing (**Figure 2C**). This precludes deductions on the potential effects of helminths on other constituents of the gut microbiome such as fungi and viruses. Of the 19 studies analyzed, the V3-V4 region (35%) and V4 region (30%) were the most targeted regions for the 16S rRNA microbiome sequencing. We analyzed all these studies irrespective of what region was used for sequencing and report an overall effect of helminth infection on the gut microbiome.

### Distribution of studies by type of helminth infection

Most of the individuals analyzed in these articles were infected with more than one helminth (57.9%), while the second largest proportion (10.5%) was infected with *Schistosoma haematobium* only (**Figure 2B**). Any studies that did not fall into these two categories examined individuals with singular infections of different worms. *S. mansoni* is *Schistosoma mansoni*, *S. haematobium* is *Schistosoma haematobium*, *S. stercoralis* is *Strongyloides stercoralis*, *N. americanus* is *Necator americanus*, *E. vermicularis* is *Enterobius vermicularis*, *Clonorchis sinensis* and *H. taichui* is *Haplorchis taichui*.

### Helminth infection is associated with increased alpha diversity

**Figure 5:**
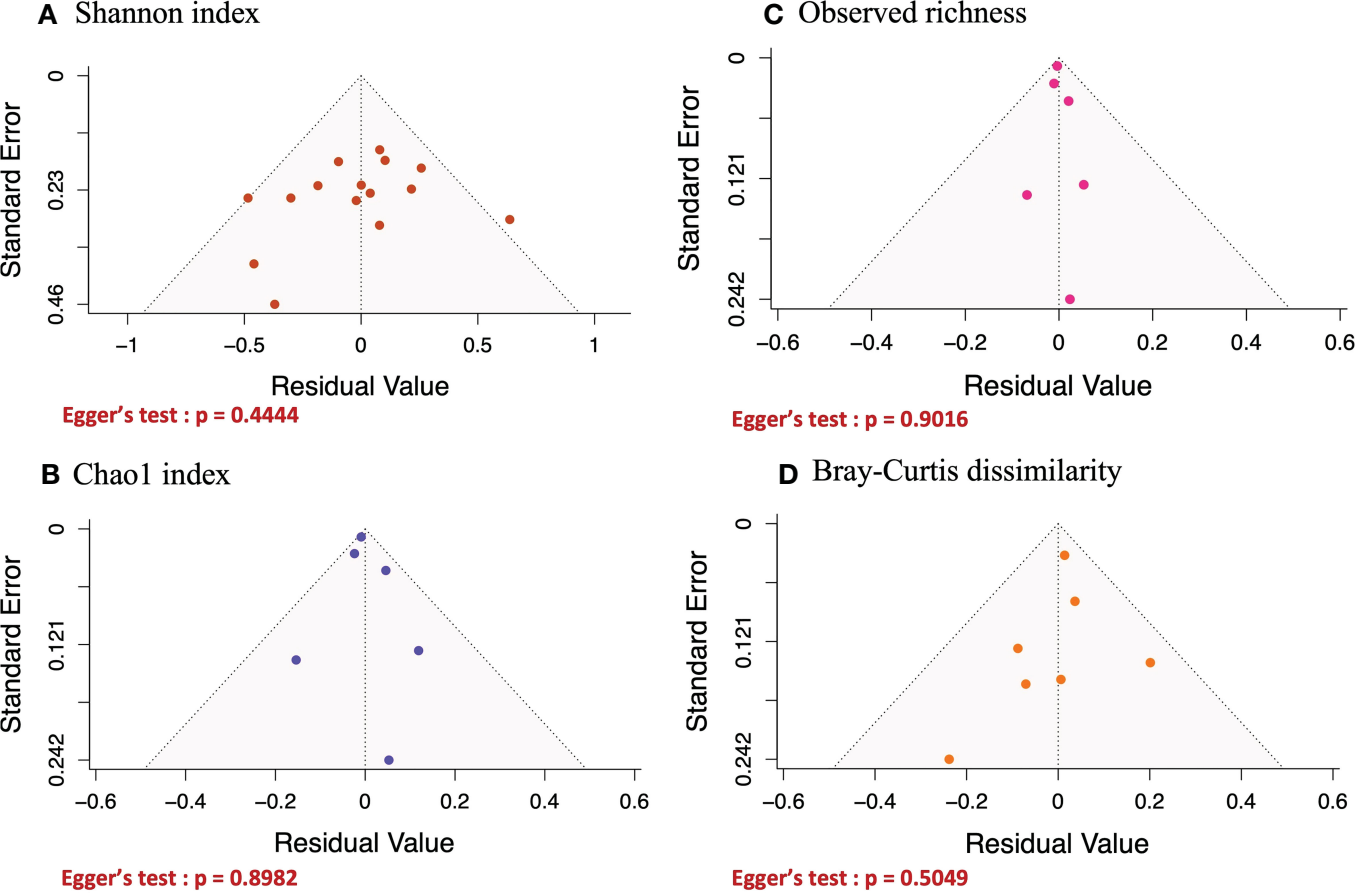
Funnel plots of the effect of helminth infections on Shannon **(A)** and Chao1 **(B)** indices, and on observed richness **(C)** and Bray-curtis dissimilarity **(D)**. Egger’s test was performed on all funnel plots and their P.values were > 0.05, implying that none of the results generated in **Figure 3** above was affected by publication bias.

Funnel plot and Egger’s tests were performed to assess publication bias for any of the metrics being analyzed (**Figure 5**). For all the diversity metrics, the p-values from Egger’s tests were greater than 0.05, indicating that there was no publication bias in our meta-analysis results. One study (Miguel et al.) was eliminated because it had too small sample size (n=6) to be included in our analysis.

On calculating the average Shannon index for all datasets obtained from the selected articles, we observed 10 out of 15 datasets (66.7%) had a higher average Shannon index in helminth positive individuals compared to the individuals without helminth infection. For many of the examined datasets, the average Shannon index was higher in helminth positive compared to helminth negative individuals (Cooper et al., 2013; Jenkins et al., 2018; Martin et al., 2018; Rosa et al., 2018; Xu et al., 2018; Gordon et al., 2020; Rubel et al., 2020). Although, we did identify other datasets where helminth positive participants had lower average Shannon indices compared to those that were not infected with helminths (Ajibola et al., 2018; Xu et al., 2018; Easton et al., 2019). Due to the apparent discrepancy in data and results available on the effects of helminth infection on gut microbiome, we conducted a meta-analysis (using random-effects model) to assess the overall effect of helminth infection on the Shannon index, adjusting for age, sex and anthelminthic treatment. Other alpha diversity metrics including Chao1 index and observed richness were also calculated and separate meta-analyses were conducted.

**Figure 3A** shows the SMD calculated using the mean Shannon indices described above, the sample size and standard deviations, 95% confidence interval of each study and the weights of each study (in percentage), as summarized in **Table 3**. Data from 10 of the studies included in the meta-analysis had positive SMD values, with three of these having SMD values where the 95% confidence intervals excluded the null value 0. This means that the bacterial diversity was significantly higher in the helminth infected individuals compared to the uninfected in the three studies. The remaining studies had negative SMD values, but all had 95% confidence intervals including the null value 0, indicative of participants without helminth infection having no significant difference in bacterial diversity compared to those infected with helminths. Using SMD calculated from the average Shannon index of every study as the summary statistic and adjusting for sex, age, and helminth treatment, our meta-analysis results show an overall SMD, 0.17 (95% CI, 0.03 to 0.31), indicating that having helminth infections is associated with a higher gut bacterial diversity compared to being helminth-uninfected. Heterogeneity *I^2^* statistic of each of the forest plots is shown at the left bottom of the figure. Following interpretation from earlier research (Higgins and Thompson, 2002), meta-analysis results for Chao1, observed richness and BCD all had *I^2 = ^* 0% indicating that there was minimal heterogeneity between study effect estimates, while the plot for Shannon index had *I^2 = ^* 44.33%, indicating small-moderate heterogeneity between the effect estimates.

**Table 3:** Mean Shannon indices for Helminth infected and Helminth uninfected individuals across the selected studies.

	Helminth-infected			Helminth-uninfected								
Source	n1i	m1i	sd1i	n2i	m2i	sd2i	Age	male	females	Pfemales	treatment	Ptreatment
Cooper et al., 2013	64	3.06	0.67	29	3.29	0.52	10.33	48	45	48.39	0	0
Jenkins et al., 2018	20	3.57	0.47	24	3.38	0.42	72.34	26	18	40.91	13/44	29.55
Gordon et al., 2020	168	1.76	0.41	38	1.58	0.33	32.68	91	115	55.83	0	0
Ajibola et al., 2018	24	3.69	0.67	25	3.77	0.75	12.23	41	8	16.33	0	0
Easton_a et al., 2019	15	4.83	0.46	15	4.95	0.43	16.5	10	20	66.67	0	0
Easton_b et al., 2019	48	2.49	0.42	48	2.5	0.38	13.75	33	65	66.33	0	0
Martin (2008) et al., 2018	94	0.92	0.25	56	0.88	0.21	27.63	66	81	55.1	0	0
Martin (2010), et al., 2018	58	0.93	0.23	92	0.92	0.21	29.63	66	81	55.1	69/150	46
Rosa (Indonesia 2010) et al., 2018	50	3.02	0.27	83	2.96	0.32	27.3	59	74	55.64	66/133	49.62
Rosa (Indonesia 2008) et al., 2018	78	3.02	0.31	43	2.96	0.32	26	54	67	55.37	0	0
Martin et al., 2019	44	0.93	0.25	21	0.88	0.22	25.66	30	35	53.85	0	0
Rosa (Liberia) et al., 2018	26	2.81	0.39	48	2.45	0.45	28	34	40	54.05	0	0
Rubel et al., 2020	234	4.14	0.48	343	3.92	0.52	39.47	234	341	59.3	0	0
Xu et al. a 2018	38	3.69	0.51	34	3.88	0.51	40.43	47	25	34.72	0	0
Xu et al. b 2018	9	3.62	0.57	8	3.48	0.65	70.83	8	9	52.94	0	0

Table shows source article, number of helminth infected participants (nli), the average of Shannon index (mli) in helminth infected participants, its standard deviation (sdli), number of helminth uninfected, average of Shannon in uninfected (m2li), standard deviation (sd2i), average age of participants (Age), the number of male participants, number of female participants, percentage of females (Pfemales), numbers of participants treated (treatment) and their percentage relative to the total number of participants (Ptreatment).

### Helminth infection is associated with increased bacterial richness

On analyzing average observed richness and combining studies according to similar taxa level, all the studies had higher observed richness in helminth infected participants compared to those without infection. The SMDs for observed richness from the random effects model meta-analysis (**Figure 3B**) of 6 datasets (based on same taxa-level) were all positive values and their respective confidence intervals included the null value 0, indicating that there was no significant difference in the observed bacterial richness when each study was analyzed separately. However, when all studies are combined in a meta-analysis and adjusting for sex, age, and helminth treatment, an overall SMD of 0.25 (95% CI, 0.04 to 0.41) was observed; therefore, indicating that helminth infection is associated with increased bacterial richness compared to having no helminth infection.

Similarly, the Chao1 index SMDs (**Figure 3C**) of six studies (based on same taxa-level) were all positive values with 95% confidence intervals including the null value 0. When all these studies were combined into a metanalysis to assess the overall effect of helminth infection on bacterial richness (after adjustment for sex, age, and helminth treatment), we found that helminth infection was associated with increased bacterial richness (SMD overall effect, 0.23; 95% CI, 0.07 to 0.43).

### Helminth infection and beta-diversity

Using a similar approach to how we established changes in alpha diversity metrics in the gut bacteria microbiome and helminth infections, we performed a similar analysis to explore the differences in the average between-individual beta diversity of the helminth-infected and -uninfected groups. As a metric for beta-diversity, BCD of helminth infected and uninfected groups was calculated. On analyzing average BCD and adjusting for sex, age, and helminth treatment, the overall effect SMD from the random effects meta-analysis was positive, but the 95% confidence interval included the null value 0. (SMD, 0.08; 95% CI, -0.11 to 0.28, **Figure 3D**). This means that the average between-individual beta diversity of the infected and uninfected groups in our analysis are similar, but the richness and relative abundancies of these taxa are significantly different in individuals with helminth infections (compared to uninfected individuals) as shown by the alpha-diversity metrics.

## Discussion

Our results illustrate that it is possible to conduct an informative meta-analysis highlighting the relationship between helminth infection and bacterial diversity and richness, by calculating the average alpha diversity metrics including Shannon, observed richness, Chao1 and Simpson indices, and beta-diversity using BCD. We show that infection with helminths is associated with an increased bacterial diversity, as shown by an overall adjusted effect of 0.17(95% CI,0.03 to 0.3) for Shannon index and increased richness indicated by an overall adjusted effect of 0.23 (95% CI,0.04 to 0.41) for observed richness and 0.25 (95% CI,0.07 to 0.43) for Chao1 index. These results are consistent with findings from a recent meta-analysis that used a different methodological approach (merging raw sequences from the selected studies) to evaluate the impact of helminth infection on the gut microbiome (Kupritz et al., 2021). They found an increase in alpha diversity (Shannon, InvSimpson indices) after adjusting for age, and in bacterial richness (Chao1 index) for individuals infected with multiple helminths compared to those that are not infected.

Chronic helminths infection is known to influence host immune responses to infections such as Malaria (Hartgers and Yazdanbakhsh, 2006), Tuberculosis (Cadmus et al., 2020), vaccines (Nkurunungi et al., 2021; Natukunda et al., 2022), and in diseases like cardiovascular disease (Sanya et al., 2020), autoimmune disease (Elliott et al., 2003), and allergy (Mpairwe et al., 2011). Our analysis and results, therefore, bring us closer to understanding whether helminths modulate host susceptibility to other pathogens and diseases such as cardiovascular disease, in part, through the gut microbiome changes that they may induce. This is further accentuated by research that has implicated the changes in the host’s gut microbiota in autoimmune diseases such as inflammatory bowel disease (Ramanan et al., 2016) and rheumatoid arthritis (Picchianti-Diamanti et al., 2018). As such, illustrating that helminths can alter the gut microbiome may be a key step in deciphering the role of the helminth parasites in disease outcomes, immunity to infection and non-communicable diseases such as cardiovascular disease as well as phenotypes such as vaccine responses.

It is noteworthy that the aforementioned results were produced from a meta-analytical approach using data for several published studies, where we re-calculated the diversity metrics such as Shannon, observed richness, Chao1 and BCD for the helminth infected and uninfected participants, obtained their respective averages and standard deviations, and combined this data into a random effects model. We opted to do average diversity metrics, as it has been used to describe and compare bacterial diversity and richness between groups in earlier meta-analyses involving microbiome data, albeit in non-helminth studies (Sze and Schloss, 2016). In addition, since there was variation in library size of the selected studies, this methodological approach critically circumvents the need to rarefy all sequences from multiple sequencing studies to an equal library size; as this would reduce power to identify the true magnitude of the diversity metrics of the groups being compared (Willis, 2019). To our knowledge, this review is the first to collect and integrate raw data from published papers on how helminth infection impacts the human gut microbiome; and analyze the collective data using average alpha- and beta-diversity measures in a random-effects model. Previous research has been done by analyzing all sequences as though they come from a single study, an approach that involves using subjective parameters during data processing steps such as rarefying and denoising that are known to influence or undermine the power of the overall result estimates. However, our approach that we present here entails merging average diversity metrics from individual microbiome studies and as such circumvents this caveat faced by earlier studies and makes it more meaningful for microbiome analysis. We envisage that our approach is integral in standardizing microbiome research through supporting meta-analyses. All selected articles span each continent, and therefore gives us confidence that our results are representative of all populations across the globe. As the highest numbers of helminth infections (exposure in our analysis) occur in SSA and Asia, it is meaningful that most of the articles included in this systematic review involve more participants from those regions compared to other continents with lower incidence of helminth infections (for example Europe). Despite this fact, the number of articles from Asia was greater than those from SSA, suggesting that the high helminth infection rates of SSA is not the sole driver for researchers investigating the importance of helminth infections and the gut microbiome.

From our study, we also note that the selected articles investigating helminth-gut microbiome associations from different geographical locations. For example, we show a disproportionately low number of articles from SSA (**Figure 4**). This may be because African populations are generally underrepresented in human microbiome studies due to factors such as insufficient capacity building and a lack of bioinformatic resources to enable the analysis of microbiome data even with collaborative initiatives such as H3A Africa (Allali et al., 2021) in place.

In addition to differences in the type of helminths, studies also varied depending on whether the participants had either single or multiple worm infections. It is possible that different helminths and stages of infection may have differing effects on the human gut microbiome, given that they occupy different niches within the host and induce a range of different immunological effects that could be systemic and/or local. In the current study, it was not possible to evaluate the effect of specific worm species on the gut microbiome in our meta-analysis because many study participants from the selected studies were infected with more than one helminth species. Importantly, one of the strengths of our study is that the estimates of heterogeneity (shown in **Figure 3**) for all the diversity metrices were low, indicating that the observed effects of helminth infection on diversity and richness across all the selected studies were consistent.

We further highlight several factors that we think could be important to consider when conducting future research aimed at investigating the interactions between helminths and gut microbiome in humans. For example; it is important to note is that helminth infection status across all the studies was based on PCR and microscopy, which are tests for current helminth infection, yet, it is possible that participants without current helminth infection may still evoke an immune response profile from previous helminth exposure, which could in turn influence the gut microbiome profile of these individuals to resemble more closely those with current helminth infection. Lack of this information from the original studies on previous helminths exposure and/or the difference in the methods used to assess helminth infection across all studies may also contribute to variation and misclassification of individuals as positive or negative. A few of the studies included in this review involved participants that were subjected to anthelminthic treatment, however since the number of original studies that collected this data is too small, there was not enough data to investigate how anthelminthic treatment may alter the relationship between the gut microbiome and the host immune response.

Further, the hypervariable 16S rRNA region that was used to profile the fecal microbiome differs from one study to another, and the studies were all based on 16S rRNA sequencing. This implies that our meta-analysis focused on only bacteria-based studies. Although bacteria are the major constituents of the gut microbiota, it would be of additional importance to gather more data on the virome and mycobiome from helminth infected individuals in the future because both have potential to cause and influence disease (Pfeiffer and Virgin, 2016). Diet is a source of substrate not only for the host but for the gut microbes too (Bolte et al., 2021), as such, the consistent diet of an individual can shape their gut microbial diversity and composition. Earlier research has shown that diet could have a greater effect on beta-diversity than on alpha diversity in humans and wild animals. Further, individuals living in urban areas have different gut microbial profiles from those living in rural areas (Oduaran et al., 2020). We envisage that individuals in our study come from similar environments and hence might have similar diets, partly explaining why average between-individual beta diversity did not differ significantly between the two groups in our analysis (Li et al., 2016; Xiao et al., 2022). However, in order to improve our interpretation of similar systematic reviews and analyses in the future, data on the dominant diet (collected by methods such as nutritional questionnaires and next generation sequencing technologies) (Pompanon et al., 2012; Lee et al., 2019) and residence of participants should be collected and availed by original articles. This data would then allow deeper meta-analysis accounting for these factors in the model of analysis as a possible source of confounding.

Furthermore, there has been an increase in evidence of circadian rhythms in intestinal microbiota from multiple human and experimental mouse studies (Leone et al., 2015; Liang et al., 2015; Paulose et al., 2016) and it would be interesting to assess how the time of sample collection would affect the helminth-microbiome associations observed. Therefore, going forward we recommend that studies report the time of collection of samples to allow future helminth-microbiome random model meta-analyses to incorporate time of collection.

Lastly, we reckon that information on antibiotic use by participants would be useful for future analyses. This is because earlier research has shown that antibiotics reduce bacterial diversity, and may facilitate colonization by a particular species over others (Lange et al., 2016). Palleja et al. showed that a combined administration of gentamicin, vancomycin and meropenem was associated with an increase in Enterobacteriaceae and a decrease in Bifidobacteria (Palleja et al., 2018). We therefore propose that since antibiotics can have an impact on the gut microbiome, data on antibiotics usage in participants with or without helminth infection would be valuable. Incorporating such data into meta-analysis models and accounting for antibiotic use would improve the power of the studies investigating the effect of helminths on the gut microbiome in humans.

## Conclusion

Using a novel methodological approach, our study, within its acknowledged limitations, has shown that helminth infection is correlated with increase in one’s bacterial diversity and richness. These results lay foundation for possible new insights on how the helminths may alter susceptibility to infection, disease, and immune response to vaccines, by inducing changes in the gut microbiome of the host. Further, we note that it is uncommon for studies to report average values of diversity/richness metrics, making it laborsome to recalculate the means and standard deviations of the metrics from raw data files for all publications that could be a part of a systematic review. We therefore recommend that original research articles report the averages and standard deviations of metrics of microbial diversity in their results, to allow for more meta-analyses and comparability of studies investigating the effects of helminth on gut microbiome in the future. We also review how factors such as diet, environment, time of sample collection and parasitology tests could impact results from studies investigating the impact of helminths and gut microbiome. Given the heterogeneity of effects that helminths may have on humans’ gut microbiome, our study reports the overall effect of helminths on microbiome and proposes a random effects model that may be vital in analyzing the helminth-microbiome interactions.

## Author contributions

BW, AME, DPK, and RKG: design and conceptualization. BW and ML: literature search. BW, JN, and ELW: data analysis. BW: drafting the initial manuscript. BW, AME, DPK, RKG, MAEL, JN, and ELW revised and edited the manuscript, and approved the submitted version.
