# Supplementary material for: The effects of helminth infections on the human gut microbiome: a systematic review and meta-analysis
Source: Front Microbiomes. 2023 May 18;2:1174034. doi: 10.3389/frmbi.2023.1174034 (PMC12993577; doi:10.3389/frmbi.2023.1174034)
Supplement: Supplementary file 1 [file DataSheet_1.docx]

**Appendix S2: ICROMS Quality Assessment Tool**
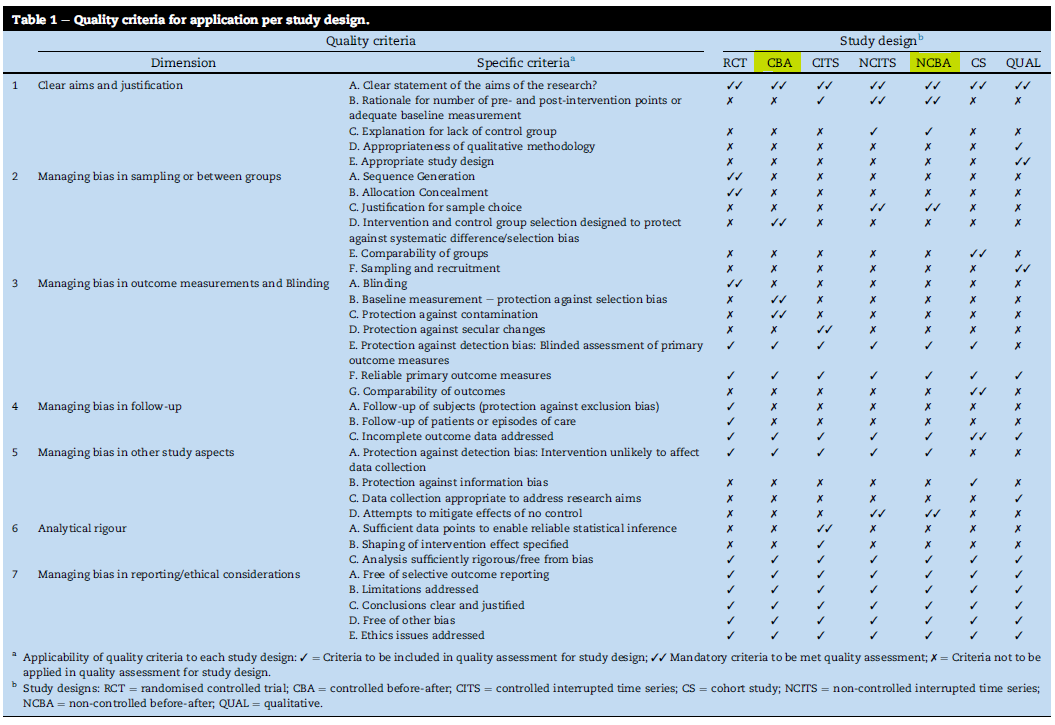


Key notes:

- For the NCBA section, the maximum available score for a study is 30 points (minimum score required: 18) and for the CS section, the maximum available score is 28 and for the CBA section, the maximum score is 28 (minimum score for both CBA and CS: 17).
- ICROM requires that studies that are unable to meet the minimum required score or meet mandatory criteria must not be included in the review.

# **Results of Quality Assessment using ICROMS**

## Study title/Author: (NCBA)

| **Category** | **Yes (1)/No (0)/Unsure (U)** |
| --- | --- |
| 1. **Clear aims and justification** | |
| a. Clear statement of the aims of the research? * |  |
| b. Rationale for number of pre- and post-intervention points or adequate baseline measurement* |  |
| c. Explanation for lack of control group |  |
| 1. **Managing bias in sampling or between groups** | |
| c. Justification of sample choice* |  |
| 1. **Managing bias in outcome measurements and blinding** | |
| e. Protection against detection bias: Blinded assessment of primary outcome measures |  |
| f. Reliable primary outcome measures |  |
| 1. **Managing bias in follow-up** | |
| c. Incomplete outcome data addressed |  |
| 1. **Managing bias in other study aspects** | |
| a. Protection against detection bias: Intervention unlikely to affect data collection |  |
| d. Attempts to mitigate effects of no control* |  |
| 1. **Analytical rigour** | |
| c. Analysis sufficiently rigorous/free from bias |  |
| 1. **Managing bias in reporting/ethical consideration** | |
| a. Free of selective outcome reporting |  |
| b. Limitations addressed |  |
| c. Conclusions clear and justified |  |
| d. Free of other bias |  |
| e. Ethics issues addressed |  |
| **TOTAL:** |  |

Study / Author: (CS)

| **Category** | **Yes (2)/No (0)/Unsure (1)** |
| --- | --- |
| 1. **Clear aims and justification** | |
| a. Clear statement of the aims of the research? * |  |
| 1. **Managing bias in sampling or between groups** | |
| E. comparability of groups* |  |
| 1. **Managing bias in outcome measurements and blinding** | |
| e. Protection against detection bias: Blinded assessment of primary outcome measures |  |
| f. Reliable primary outcome measures |  |
| G. comparability of outcomes* |  |
| 1. **Managing bias in follow-up** | |
| c. Incomplete outcome data addressed |  |
| 1. **Managing bias in other study aspects** | |
| b. Protection against information bias |  |
| d. Attempts to mitigate effects of no control* |  |
| 1. **Analytical rigour** | |
| c. Analysis sufficiently rigorous/free from bias |  |
| 1. **Managing bias in reporting/ethical consideration** | |
| a. Free of selective outcome reporting |  |
| b. Limitations addressed |  |
| c. Conclusions clear and justified |  |
| d. Free of other bias |  |
| e. Ethics issues addressed |  |
| **TOTAL:** |  |

*Mandatory criterion – Fail if not fulfilled.

Study/Author: (CBA)

| **Category** | **Yes (2)/No (0)/Unsure (1)** |
| --- | --- |
| 1. **Clear aims and justification** | |
| a. Clear statement of the aims of the research? * |  |
| 1. **Managing bias in sampling or between groups** | |
| d. Intervention and control group selection designed to protect against systematic difference/selection bias* |  |
| 1. **Managing bias in outcome measurements and blinding** | |
| b. Baseline measurement - protection against selection bias* |  |
| c. Protection against contamination* |  |
| e. Protection against detection bias: Blinded assessment of primary outcome measures |  |
| f. Reliable primary outcome measures |  |
| 1. **Managing bias in follow-up** | |
| c. Incomplete outcome data addressed |  |
| 1. **Managing bias in other study aspects** | |
| a. Protection against detection bias: Intervention unlikely to affect data collection |  |
| 1. **Analytical rigour** | |
| c. Analysis sufficiently rigorous/free from bias |  |
| 1. **Managing bias in reporting/ethical consideration** | |
| a. Free of selective outcome reporting |  |
| b. Limitations addressed |  |
| c. Conclusions clear and justified |  |
| d. Free of other bias |  |
| e. Ethics issues addressed |  |
| **TOTAL:** |  |

*Mandatory criterion – Fail if not fulfilled.

CS Format: Jenkins *et al*. A comprehensive analysis of the faecal microbiome and

metabolome of Strongyloides stercoralis infected volunteers from

## a non-endemic area, 2018.

| **Category** | **Yes (2)/No (0)/Unsure (1)** |
| --- | --- |
| 1. **Clear aims and justification** | |
| a. Clear statement of the aims of the research? * | 2 |
| 1. **Managing bias in sampling or between groups** | |
| E. comparability of groups* | 2 |
| 1. **Managing bias in outcome measurements and blinding** | |
| e. Protection against detection bias: Blinded assessment of primary outcome measures | 2 |
| f. Reliable primary outcome measures | 2 |
| G. comparability of outcomes* | 2 |
| 1. **Managing bias in follow-up** | |
| c. Incomplete outcome data addressed | 1 |
| 1. **Managing bias in other study aspects** | |
| b. Protection against information bias | 1 |
| d. Attempts to mitigate effects of no control* | 2 – since they included controls |
| 1. **Analytical rigour** | |
| c. Analysis sufficiently rigorous/free from bias | 2 |
| 1. **Managing bias in reporting/ethical consideration** | |
| a. Free of selective outcome reporting | 1 |
| b. Limitations addressed | 2 |
| c. Conclusions clear and justified | 2 |
| d. Free of other bias | 1 |
| e. Ethics issues addressed | 2 |
| **TOTAL:** | 24 |

*Mandatory criterion – Fail if not fulfilled.

CS Format: Jenkins *et al*, Infections by human gastrointestinal helminths are associated with changes in faecal microbiota diversity and composition, 2017.

| **Category** | **Yes (2)/No (0)/Unsure (1)** |
| --- | --- |
| 1. **Clear aims and justification** | |
| a. Clear statement of the aims of the research? * | 2 |
| 1. **Managing bias in sampling or between groups** | |
| E. comparability of groups* | 2 |
| 1. **Managing bias in outcome measurements and blinding** | |
| e. Protection against detection bias: Blinded assessment of primary outcome measures | 2 |
| f. Reliable primary outcome measures | 2 |
| G. comparability of outcomes* | 2 |
| 1. **Managing bias in follow-up** | |
| c. Incomplete outcome data addressed | 1 |
| 1. **Managing bias in other study aspects** | |
| b. Protection against information bias | 2 |
| d. Attempts to mitigate effects of no control* | 2 – they included controls |
| 1. **Analytical rigour** | |
| c. Analysis sufficiently rigorous/free from bias | 2 |
| 1. **Managing bias in reporting/ethical consideration** | |
| a. Free of selective outcome reporting | 2 |
| b. Limitations addressed | 2 |
| c. Conclusions clear and justified | 1 |
| d. Free of other bias | 1 |
| e. Ethics issues addressed | 2 |
| **TOTAL:** | 25 |

## *Mandatory criterion – Fail if not fulfilled.

CBA Format: Prommi *et al*: Intestinal parasites in rural communities in Nan Province, Thailand:

## Changes in bacterial gut microbiota associated with minute intestinal fluke infection, 2020

| **Category** | **Yes (2)/No (0)/Unsure (1)** |
| --- | --- |
| 1. **Clear aims and justification** | |
| a. Clear statement of the aims of the research? * | 2 |
| 1. **Managing bias in sampling or between groups** | |
| d. Intervention and control group selection designed to protect against systematic difference/selection bias* | 2 |
| 1. **Managing bias in outcome measurements and blinding** | |
| b. Baseline measurement - protection against selection bias* | 2 |
| c. Protection against contamination* | 2 |
| e. Protection against detection bias: Blinded assessment of primary outcome measures | 1 |
| f. Reliable primary outcome measures | 2 |
| 1. **Managing bias in follow-up** | |
| c. Incomplete outcome data addressed | 2 |
| 1. **Managing bias in other study aspects** | |
| a. Protection against detection bias: Intervention unlikely to affect data collection | 2 |
| 1. **Analytical rigour** | |
| c. Analysis sufficiently rigorous/free from bias | 1 |
| 1. **Managing bias in reporting/ethical consideration** | |
| a. Free of selective outcome reporting | 2 |
| b. Limitations addressed | 1 |
| c. Conclusions clear and justified | 1 |
| d. Free of other bias | 2 |
| e. Ethics issues addressed | 2 |
| **TOTAL:** | 24 |

*Mandatory criterion – Fail if not fulfilled.

CBA Format: Schneeberger *et al*, Investigations on the interplays between Schistosoma mansoni, praziquantel and the gut microbiome, 2018

| **Category** | **Yes (2)/No (0)/Unsure (1)** |
| --- | --- |
| 1. **Clear aims and justification** | |
| a. Clear statement of the aims of the research? * | 2 |
| 1. **Managing bias in sampling or between groups** | |
| d. Intervention and control group selection designed to protect against systematic difference/selection bias* | 2 |
| 1. **Managing bias in outcome measurements and blinding** | |
| b. Baseline measurement - protection against selection bias* | 2 |
| c. Protection against contamination* | 2 |
| e. Protection against detection bias: Blinded assessment of primary outcome measures | 2 |
| f. Reliable primary outcome measures | 2 |
| 1. **Managing bias in follow-up** | |
| c. Incomplete outcome data addressed | 1 |
| 1. **Managing bias in other study aspects** | |
| a. Protection against detection bias: Intervention unlikely to affect data collection | 2 |
| 1. **Analytical rigour** | |
| c. Analysis sufficiently rigorous/free from bias | 2 |
| 1. **Managing bias in reporting/ethical consideration** | |
| a. Free of selective outcome reporting | 1 |
| b. Limitations addressed | 1 |
| c. Conclusions clear and justified | 2 |
| d. Free of other bias | 1 |
| e. Ethics issues addressed | 2 |
| **TOTAL:** | 24 |

*Mandatory criterion – Fail if not fulfilled.

CS Format: Rosa *et al*, Differential human gut microbiome assemblages during soil-transmitted helminth infections in Indonesia and Liberia, 2018

| **Category** | **Yes (2)/No (0)/Unsure (1)** |
| --- | --- |
| 1. **Clear aims and justification** | |
| a. Clear statement of the aims of the research? * | 2 |
| 1. **Managing bias in sampling or between groups** | |
| E. comparability of groups* | 2 |
| 1. **Managing bias in outcome measurements and blinding** | |
| e. Protection against detection bias: Blinded assessment of primary outcome measures | 2 |
| f. Reliable primary outcome measures | 2 |
| G. comparability of outcomes* | 2 |
| 1. **Managing bias in follow-up** | |
| c. Incomplete outcome data addressed | 1 |
| 1. **Managing bias in other study aspects** | |
| b. Protection against information bias | 1 |
| d. Attempts to mitigate effects of no control* | 2 – they included controls |
| 1. **Analytical rigour** | |
| c. Analysis sufficiently rigorous/free from bias | 2 |
| 1. **Managing bias in reporting/ethical consideration** | |
| a. Free of selective outcome reporting | 2 |
| b. Limitations addressed | 1 |
| c. Conclusions clear and justified | 2 |
| d. Free of other bias | 1 |
| e. Ethics issues addressed | 2 |
| **TOTAL:** | 24 |

## *Mandatory criterion – Fail if not fulfilled

CS Format: Rubel *et al,* Lifestyle and the presence of helminths is associated with gut microbiome composition in Cameroonians, 2020.

| **Category** | **Yes (2)/No (0)/Unsure (1)** |
| --- | --- |
| 1. **Clear aims and justification** | |
| a. Clear statement of the aims of the research? * | 2 |
| 1. **Managing bias in sampling or between groups** | |
| E. comparability of groups* | 2 |
| 1. **Managing bias in outcome measurements and blinding** | |
| e. Protection against detection bias: Blinded assessment of primary outcome measures | 2 |
| f. Reliable primary outcome measures | 2 |
| G. comparability of outcomes* | 2 |
| 1. **Managing bias in follow-up** | |
| c. Incomplete outcome data addressed | 1 |
| 1. **Managing bias in other study aspects** | |
| b. Protection against information bias | 2 |
| d. Attempts to mitigate effects of no control* | 2 |
| 1. **Analytical rigour** | |
| c. Analysis sufficiently rigorous/free from bias | 2 |
| 1. **Managing bias in reporting/ethical consideration** | |
| a. Free of selective outcome reporting | 1 |
| b. Limitations addressed | 1 |
| c. Conclusions clear and justified | 2 |
| d. Free of other bias | 1 |
| e. Ethics issues addressed | 2 |
| **TOTAL:** | 24 |

## *Mandatory criterion – Fail if not fulfilled

## NCBA format: Cantacessi *et al* Impact of experimental hookworm infection on the gut human gut microbiota, 2020

| **Category** | **Yes (1)/No (0)/Unsure (U)** |
| --- | --- |
| 1. **Clear aims and justification** | |
| a. Clear statement of the aims of the research? | 2 |
| b. Rationale for number of pre- and post-intervention points or adequate baseline measurement | 1 |
| c. Explanation for lack of control group | 0 |
| 1. **Managing bias in sampling or between groups** | |
| c. Justification of sample choice | 1 |
| 1. **Managing bias in outcome measurements and blinding** | |
| e. Protection against detection bias: Blinded assessment of primary outcome measures | 2 |
| f. Reliable primary outcome measures | 2 |
| 1. **Managing bias in follow-up** | |
| c. Incomplete outcome data addressed | 1 |
| 1. **Managing bias in other study aspects** | |
| a. Protection against detection bias: Intervention unlikely to affect data collection | 2 |
| d. Attempts to mitigate effects of no control | 0 |
| 1. **Analytical rigour** | |
| c. Analysis sufficiently rigorous/free from bias | 2 |
| 1. **Managing bias in reporting/ethical consideration** | |
| a. Free of selective outcome reporting | 2 |
| b. Limitations addressed | 0 |
| c. Conclusions clear and justified | 1 |
| d. Free of other bias | 0 |
| e. Ethics issues addressed | 2 |
| **TOTAL:** | 18 |

CBA format: Cooper *et al,* Patent Human Infections with the Whipworm, Trichuris

trichiura, Are Not Associated with Alterations in the faecal microbiota, 2013.

| **Category** | **Yes (1)/No (0)/Unsure (U)** |
| --- | --- |
| 1. **Clear aims and justification** | |
| a. Clear statement of the aims of the research? | 2 |
| 1. **Managing bias in sampling or between groups** | |
| d. Intervention and control group selection designed to protect against systematic difference/selection bias | 2 |
| 1. **Managing bias in outcome measurements and blinding** | |
| b. Baseline measurement - protection against selection bias | 1 |
| c. Protection against contamination | 1 |
| e. Protection against detection bias: Blinded assessment of primary outcome measures | 2 |
| f. Reliable primary outcome measures | 2 |
| 1. **Managing bias in follow-up** | |
| c. Incomplete outcome data addressed | 0 |
| 1. **Managing bias in other study aspects** | |
| a. Protection against detection bias: Intervention unlikely to affect data collection | 2 |
| 1. **Analytical rigour** | |
| c. Analysis sufficiently rigorous/free from bias | 1 |
| 1. **Managing bias in reporting/ethical consideration** | |
| a. Free of selective outcome reporting | 0 |
| b. Limitations addressed | 1 |
| c. Conclusions clear and justified | 2 |
| d. Free of other bias | 1 |
| e. Ethics issues addressed | 2 |
| **TOTAL:** | 19 |

## CBA: Ajibola, Urogenital schistosomiasis is associated with signatures of microbiome dysbiosis in Nigerian adolescents, 2019

| **Category** | **Yes (1)/No (0)/Unsure (U)** |
| --- | --- |
| 1. **Clear aims and justification** | |
| a. Clear statement of the aims of the research? | 2 |
| 1. **Managing bias in sampling or between groups** | |
| d. Intervention and control group selection designed to protect against systematic difference/selection bias | 2 |
| 1. **Managing bias in outcome measurements and blinding** | |
| b. Baseline measurement - protection against selection bias | 1 |
| c. Protection against contamination | 1 |
| e. Protection against detection bias: Blinded assessment of primary outcome measures | 1 |
| f. Reliable primary outcome measures | 2 |
| 1. **Managing bias in follow-up** | |
| c. Incomplete outcome data addressed | 2 |
| 1. **Managing bias in other study aspects** | |
| a. Protection against detection bias: Intervention unlikely to affect data collection | 2 |
| 1. **Analytical rigour** | |
| c. Analysis sufficiently rigorous/free from bias | 1 |
| 1. **Managing bias in reporting/ethical consideration** | |
| a. Free of selective outcome reporting | 1 |
| b. Limitations addressed | 1 |
| c. Conclusions clear and justified | 2 |
| d. Free of other bias | 0 |
| e. Ethics issues addressed | 2 |
| **TOTAL:** | 20 |

## CBA: Easton *et al*. The Impact of Anthelmintic Treatment on Human Gut Microbiota Based on Cross-Sectional and Pre- and Postdeworming Comparisons in Western Kenya, 2019.

| **Category** | **Yes (1)/No (0)/Unsure (U)** |
| --- | --- |
| 1. **Clear aims and justification** | |
| a. Clear statement of the aims of the research? | 2 |
| 1. **Managing bias in sampling or between groups** | |
| d. Intervention and control group selection designed to protect against systematic difference/selection bias | 2 |
| 1. **Managing bias in outcome measurements and blinding** | |
| b. Baseline measurement - protection against selection bias | 2 |
| c. Protection against contamination | 2 |
| e. Protection against detection bias: Blinded assessment of primary outcome measures | 2 |
| f. Reliable primary outcome measures | 2 |
| 1. **Managing bias in follow-up** | |
| c. Incomplete outcome data addressed | 0 |
| 1. **Managing bias in other study aspects** | |
| a. Protection against detection bias: Intervention unlikely to affect data collection | 2 |
| 1. **Analytical rigour** | |
| c. Analysis sufficiently rigorous/free from bias | 2 |
| 1. **Managing bias in reporting/ethical consideration** | |
| a. Free of selective outcome reporting | 2 |
| b. Limitations addressed | 1 |
| c. Conclusions clear and justified | 2 |
| d. Free of other bias | 1 |
| e. Ethics issues addressed | 2 |
| **TOTAL:** | 23 |

CBA. Catherine A *et al*. Helminths, polyparasitism, and the gut microbiome in the Philippines, 2020.

| **Category** | **Yes (1)/No (0)/Unsure (U)** |
| --- | --- |
| 1. **Clear aims and justification** | |
| a. Clear statement of the aims of the research? | 2 |
| 1. **Managing bias in sampling or between groups** | |
| d. Intervention and control group selection designed to protect against systematic difference/selection bias | 2 |
| 1. **Managing bias in outcome measurements and blinding** | |
| b. Baseline measurement - protection against selection bias | 2 |
| c. Protection against contamination | 2 |
| e. Protection against detection bias: Blinded assessment of primary outcome measures | 2 |
| f. Reliable primary outcome measures | 2 |
| 1. **Managing bias in follow-up** | |
| c. Incomplete outcome data addressed | 1 |
| 1. **Managing bias in other study aspects** | |
| a. Protection against detection bias: Intervention unlikely to affect data collection | 2 |
| 1. **Analytical rigour** | |
| c. Analysis sufficiently rigorous/free from bias | 2 |
| 1. **Managing bias in reporting/ethical consideration** | |
| a. Free of selective outcome reporting | 1 |
| b. Limitations addressed | 1 |
| c. Conclusions clear and justified | 2 |
| d. Free of other bias | 1 |
| e. Ethics issues addressed | 2 |
| **TOTAL:** | 22 |

CBA: Huwe *et al*, Interactions between Parasitic Infections and the Human Gut Microbiome in Odisha, India, 2019.

| **Category** | **Yes (1)/No (0)/Unsure (U)** |
| --- | --- |
| 1. **Clear aims and justification** | |
| a. Clear statement of the aims of the research? | 2 |
| 1. **Managing bias in sampling or between groups** | |
| d. Intervention and control group selection designed to protect against systematic difference/selection bias | 1 |
| 1. **Managing bias in outcome measurements and blinding** | |
| b. Baseline measurement - protection against selection bias | 1 |
| c. Protection against contamination | 2 |
| e. Protection against detection bias: Blinded assessment of primary outcome measures | 2 |
| f. Reliable primary outcome measures | 2 |
| 1. **Managing bias in follow-up** | |
| c. Incomplete outcome data addressed | 1 |
| 1. **Managing bias in other study aspects** | |
| a. Protection against detection bias: Intervention unlikely to affect data collection | 2 |
| 1. **Analytical rigour** | |
| c. Analysis sufficiently rigorous/free from bias | 1 |
| 1. **Managing bias in reporting/ethical consideration** | |
| a. Free of selective outcome reporting | 1 |
| b. Limitations addressed | 1 |
| c. Conclusions clear and justified | 2 |
| d. Free of other bias | 1 |
| e. Ethics issues addressed | 2 |
| **TOTAL:** | 21 |

CBA Format: Toro-Londono *et al*, Intestinal parasitic infection alters bacterial gut microbiota in children, 2019.

| **Category** | **Yes (2)/No (0)/Unsure (1)** |
| --- | --- |
| 1. **Clear aims and justification** | |
| a. Clear statement of the aims of the research? * | 2 |
| 1. **Managing bias in sampling or between groups** | |
| d. Intervention and control group selection designed to protect against systematic difference/selection bias* | 2 |
| 1. **Managing bias in outcome measurements and blinding** | |
| b. Baseline measurement - protection against selection bias* | 2 |
| c. Protection against contamination* | 2 |
| e. Protection against detection bias: Blinded assessment of primary outcome measures | 2 |
| f. Reliable primary outcome measures | 2 |
| 1. **Managing bias in follow-up** | |
| c. Incomplete outcome data addressed | 2 |
| 1. **Managing bias in other study aspects** | |
| a. Protection against detection bias: Intervention unlikely to affect data collection | 2 |
| 1. **Analytical rigour** | |
| c. Analysis sufficiently rigorous/free from bias | 2 |
| 1. **Managing bias in reporting/ethical consideration** | |
| a. Free of selective outcome reporting | 1 |
| b. Limitations addressed | 1 |
| c. Conclusions clear and justified | 2 |
| d. Free of other bias | 1 |
| e. Ethics issues addressed | 2 |
| **TOTAL:** | 25 |

*Mandatory criterion – Fail if not fulfilled.

CS: Kay LG et al, Differences in the Faecal Microbiome in Schistosoma haematobium Infected Children vs. Uninfected Children

| **Category** | **Yes (2)/No (0)/Unsure (1)** |
| --- | --- |
| 1. **Clear aims and justification** | |
| a. Clear statement of the aims of the research? * | 2 |
| 1. **Managing bias in sampling or between groups** | |
| E. comparability of groups* | 2 |
| 1. **Managing bias in outcome measurements and blinding** | |
| e. Protection against detection bias: Blinded assessment of primary outcome measures | 2 |
| f. Reliable primary outcome measures | 2 |
| G. comparability of outcomes* | 2 |
| 1. **Managing bias in follow-up** | |
| c. Incomplete outcome data addressed | 1 |
| 1. **Managing bias in other study aspects** | |
| b. Protection against information bias | 2 |
| d. Attempts to mitigate effects of no control* | 2 |
| 1. **Analytical rigour** | |
| c. Analysis sufficiently rigorous/free from bias | 1 |
| 1. **Managing bias in reporting/ethical consideration** | |
| a. Free of selective outcome reporting | 1 |
| b. Limitations addressed | 1 |
| c. Conclusions clear and justified | 2 |
| d. Free of other bias | 1 |
| e. Ethics issues addressed | 2 |
| **TOTAL:** | 23 |

CS: Yang et al: Impact of Enterobius vermicularis infection and mebendazole treatment on intestinal

microbiota and host immune response

| **Category** | **Yes (2)/No (0)/Unsure (1)** |
| --- | --- |
| 1. **Clear aims and justification** | |
| a. Clear statement of the aims of the research? * | 2 |
| 1. **Managing bias in sampling or between groups** | |
| E. comparability of groups* | 2 |
| 1. **Managing bias in outcome measurements and blinding** | |
| e. Protection against detection bias: Blinded assessment of primary outcome measures | 2 |
| f. Reliable primary outcome measures | 2 |
| G. comparability of outcomes* | 2 |
| 1. **Managing bias in follow-up** | |
| c. Incomplete outcome data addressed | 1 |
| 1. **Managing bias in other study aspects** | |
| b. Protection against information bias | 1 |
| d. Attempts to mitigate effects of no control* | 2 |
| 1. **Analytical rigour** | |
| c. Analysis sufficiently rigorous/free from bias | 1 |
| 1. **Managing bias in reporting/ethical consideration** | |
| a. Free of selective outcome reporting | 1 |
| b. Limitations addressed | 1 |
| c. Conclusions clear and justified | 2 |
| d. Free of other bias | 1 |
| e. Ethics issues addressed | 2 |
| **TOTAL:** | 22 |

CBA: Xu *et al*. Altered Gut Microbiota Composition in Subjects Infected with Clonorchis

## sinensis

| **Category** | **Yes (1)/No (0)/Unsure (U)** |
| --- | --- |
| 1. **Clear aims and justification** | |
| a. Clear statement of the aims of the research? | 2 |
| 1. **Managing bias in sampling or between groups** | |
| d. Intervention and control group selection designed to protect against systematic difference/selection bias | 2 |
| 1. **Managing bias in outcome measurements and blinding** | |
| b. Baseline measurement - protection against selection bias | 2 |
| c. Protection against contamination | 2 |
| e. Protection against detection bias: Blinded assessment of primary outcome measures | 2 |
| f. Reliable primary outcome measures | 2 |
| 1. **Managing bias in follow-up** | |
| c. Incomplete outcome data addressed | 1 |
| 1. **Managing bias in other study aspects** | |
| a. Protection against detection bias: Intervention unlikely to affect data collection | 2 |
| 1. **Analytical rigour** | |
| c. Analysis sufficiently rigorous/free from bias | 2 |
| 1. **Managing bias in reporting/ethical consideration** | |
| a. Free of selective outcome reporting | 2 |
| b. Limitations addressed | 1 |
| c. Conclusions clear and justified | 1 |
| d. Free of other bias | 1 |
| e. Ethics issues addressed | 2 |
| **TOTAL:** | 23 |
|  |  |

CBA: Lee et al. Helminth Colonization Is Associated with Increased Diversity of the Gut Microbiota

| **Category** | **Yes (1)/No (0)/Unsure (U)** |
| --- | --- |
| 1. **Clear aims and justification** | |
| a. Clear statement of the aims of the research? | 2 |
| 1. **Managing bias in sampling or between groups** | |
| d. Intervention and control group selection designed to protect against systematic difference/selection bias | 2 |
| 1. **Managing bias in outcome measurements and blinding** | |
| b. Baseline measurement - protection against selection bias | 2 |
| c. Protection against contamination | 1 |
| e. Protection against detection bias: Blinded assessment of primary outcome measures | 1 |
| f. Reliable primary outcome measures | 2 |
| 1. **Managing bias in follow-up** | |
| c. Incomplete outcome data addressed | 1 |
| 1. **Managing bias in other study aspects** | |
| a. Protection against detection bias: Intervention unlikely to affect data collection | 2 |
| 1. **Analytical rigour** | |
| c. Analysis sufficiently rigorous/free from bias | 2 |
| 1. **Managing bias in reporting/ethical consideration** | |
| a. Free of selective outcome reporting | 2 |
| b. Limitations addressed | 1 |
| c. Conclusions clear and justified | 2 |
| d. Free of other bias | 1 |
| e. Ethics issues addressed | 2 |
| **TOTAL:** | 22 |

CBA. Martin et al. Dynamic changes in human-gut microbiome in relation to a placebo-controlled anthelminthic trial in Indonesia

| **Category** | **Yes (1)/No (0)/Unsure (U)** |
| --- | --- |
| 1. **Clear aims and justification** | |
| a. Clear statement of the aims of the research? | 2 |
| 1. **Managing bias in sampling or between groups** | |
| d. Intervention and control group selection designed to protect against systematic difference/selection bias | 1 |
| 1. **Managing bias in outcome measurements and blinding** | |
| b. Baseline measurement - protection against selection bias | 2 |
| c. Protection against contamination | 2 |
| e. Protection against detection bias: Blinded assessment of primary outcome measures | 1 |
| f. Reliable primary outcome measures | 2 |
| 1. **Managing bias in follow-up** | |
| c. Incomplete outcome data addressed | 1 |
| 1. **Managing bias in other study aspects** | |
| a. Protection against detection bias: Intervention unlikely to affect data collection | 2 |
| 1. **Analytical rigour** | |
| c. Analysis sufficiently rigorous/free from bias | 2 |
| 1. **Managing bias in reporting/ethical consideration** | |
| a. Free of selective outcome reporting | 2 |
| b. Limitations addressed | 1 |
| c. Conclusions clear and justified | 1 |
| d. Free of other bias | 1 |
| e. Ethics issues addressed | 2 |
| **TOTAL:** | 20 |
